# Promoting inclusive metrics of success and impact to dismantle a discriminatory reward system in science

**DOI:** 10.1371/journal.pbio.3001282

**Published:** 2021-06-15

**Authors:** Sarah W. Davies, Hollie M. Putnam, Tracy Ainsworth, Julia K. Baum, Colleen B. Bove, Sarah C. Crosby, Isabelle M. Côté, Anne Duplouy, Robinson W. Fulweiler, Alyssa J. Griffin, Torrance C. Hanley, Tessa Hill, Adriana Humanes, Sangeeta Mangubhai, Anna Metaxas, Laura M. Parker, Hanny E. Rivera, Nyssa J. Silbiger, Nicola S. Smith, Ana K. Spalding, Nikki Traylor-Knowles, Brooke L. Weigel, Rachel M. Wright, Amanda E. Bates

**Affiliations:** 1 Department of Biology, Boston University, Boston, Massachusetts, United States of America; 2 Department of Biological Sciences, University of Rhode Island, Rhode Island, United States of America; 3 School of Biological Earth and Environmental Sciences, University of New South Wales, Sydney, Australia; 4 Department of Biology, University of Victoria, Victoria, British Columbia, Canada; 5 Harbor Watch, Earthplace, Inc., Westport, Connecticut, United States of America; 6 Department of Biological Sciences, Simon Fraser University, Burnaby, British Columbia, Canada; 7 The University of Helsinki, Organismal and Evolutionary Biology Research Program, Helsinki, Finland; 8 Department of Earth and Environment & Department of Biology, Boston University, Boston, Massachusetts, United States of America; 9 Department of Earth & Planetary Sciences & Bodega Marine Laboratory, University of California, Davis, California, United States of America; 10 Marine Science Center, Northeastern University, Nahant, Massachusetts, United States of America; 11 Department of Earth & Planetary Sciences & Bodega Marine Laboratory, University of California, Davis, California, United States of America; 12 School of Natural and Environmental Sciences, Newcastle University, Newcastle upon Tyne, United Kingdom; 13 Wildlife Conservation Society, Fiji Country Program, Suva, Fiji; 14 Department of Oceanography, Dalhousie University, Halifax, Nova Scotia, Canada; 15 Department of Biology, California State University, Northridge, Northridge, California, United States of America; 16 Department of Biological Sciences, Simon Fraser University, Burnaby, British Columbia, Canada; 17 School of Public Policy, College of Liberal Arts, Oregon State University, Corvallis, Oregon, United States of America; 18 Smithsonian Tropical Research Institute, Panama City, Panama; 19 University of Miami, Rosenstiel School of Marine and Atmospheric Sciences, Miami, Florida, United States of America; 20 Committee on Evolutionary Biology, University of Chicago, Chicago, Illinois, United States of America; 21 Department of Biological Sciences, Smith College, Northampton, Massachusetts, United States of America; 22 Department of Ocean Sciences, Memorial University of Newfoundland, St. John’s, New Foundland, Canada

## Abstract

Success and impact metrics in science are based on a system that perpetuates sexist and racist “rewards” by prioritizing citations and impact factors. These metrics are flawed and biased against already marginalized groups and fail to accurately capture the breadth of individuals’ meaningful scientific impacts. We advocate shifting this outdated value system to advance science through principles of justice, equity, diversity, and inclusion. We outline pathways for a paradigm shift in scientific values based on multidimensional mentorship and promoting mentee well-being. These actions will require collective efforts supported by academic leaders and administrators to drive essential systemic change.

## Abstract 2

This Essay argues that success and impact metrics in science are based on a system that perpetuates sexist and racist ‘rewards’ by prioritizing citations and impact factors; the authors advocate shifting this outdated value system to advance science through principles of justice, equity, diversity, and inclusion.

## Overview

“The most dangerous phrase in the language is: We’ve always done it this way.”

—Rear Admiral Grace Hopper

**Fig 1:**
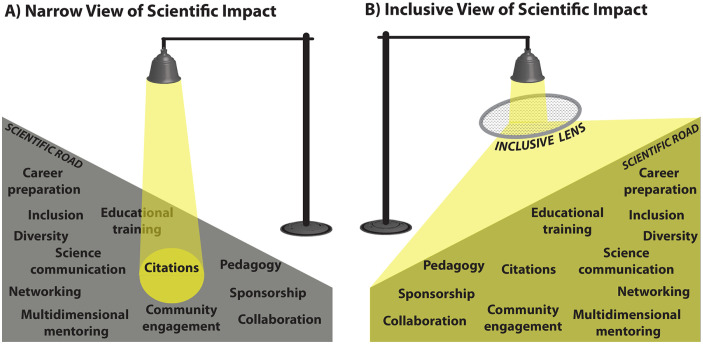
Science is suffering from observational bias in our value system. This bias is analogous to the streetlight effect where (A) citations are valued because that is where we look, despite the fact that they perpetuate gender and racial biases as metrics of success. We advocate for (B), an expanded view of success and impact that is multifaceted and includes critical areas of mentorship, inclusion, and diversity.

**Fig 2:**
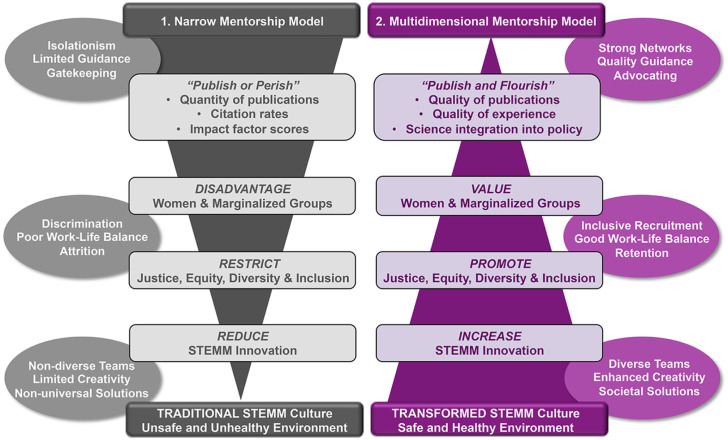
Here, we show 2 models for the disciplines of STEMM. We argue that the narrow mentorship model based on the top-down “publish or perish” approach to success and impact facilitates processes that lead to a reduction in diversity and innovation (illustrated by the inverse gray pyramid) and a detrimental STEMM culture that supports a limited subset of scholars. By contrast, a multidimensional mentorship model supported by those in leadership roles (e.g., by university and college presidents, chancellors, and provosts) working across academic institutions will incorporate diverse measures of success and impact to create system-wide change (illustrated by the purple pyramid). We argue that the latter approach can lead to increased innovation that will transform STEMM culture where processes, which support the 2 models, and outcomes of each, are side highlighted within the oval shapes. STEMM, Science, Technology, Engineering, Mathematics, and Medicine.

Experiencing challenges related to justice, equity, diversity and inclusion in science is universal across disciplines [1]. Strong evidence highlights the breadth of biases, yet action-based solutions have not been broadly adopted, and systemic change remains elusive. Under the pressure for “objective” metric-based “success” and “impact,” multiple biases are perpetuated in science. For example, flawed interpretations of data with damaging conclusions are published [2,3], including papers requiring retraction [4]. Here, our interdisciplinary, international team of women scientists publicly acknowledges and denounces the pervasive sexist and racist structures persisting within the value systems, which typify science. We further advocate to accelerate the pace of positive change in science by building on the advancements made through systemically marginalized groups, including the prior and ongoing efforts of women, Black people (we recognize this language may not be used commonly internationally and use it here to explicitly acknowledge that systemic racism disproportionately affects the lives of Black people, particularly within the United States), indigenous people, people of color, lesbian, gay, bisexual, transgender, queer, and others (LGBTQ+), and their allies (e.g., [5–9]). We (1) highlight long-standing problems associated with narrow definitions of success and impact in science; (2) advocate for expanding measures of success beyond citations to value the multifaceted nature of scientific impact (Fig 1); and (3) propose a model that values the recruitment and retention of scientists from diverse backgrounds through building safe and healthy work environments (Fig 2).

It is imperative for those holding positions of power, privilege, and visibility to take informed and strategic action rather than only engage in a performative manner. Strong actions that support justice, equity, diversity, and inclusion are essential for the accelerated evolution of the value system in science. Collectively, these changes are key to generating a greater capacity for innovation, which is essential for addressing the challenges of the present and future, such as pandemics and climate change (Fig 2).

## Pivoting the paradigm to ensure equitable evaluation in science

### (1) Citation counts are biased

One of the many detrimental constructs underpinning academic science is the “publish or perish” model that celebrates the quantity of publications, citation rates, and impact factor scores as the primary, and often sole, indicators of success and impact [10–12]. Citation metrics, which have been widely used across most research areas due to their quantitative nature and easy estimation, influence career advancement at all levels including graduate opportunities, funding success, career positions, awards, distinctions, and tenure and promotion. However, a lack of diversity among the most cited scientific authors is driven by historical demographics of faculty and those in academic leadership positions [13–16]. While there have been recent successes in increasing diversity among trainees and early-career researchers [8,9], differential recruitment, retention, and promotion rates with respect to age, sex, gender, race, and ethnicity continue to perpetuate the lack of diversity among all career levels of scientists [14,17–19]. This issue is self-perpetuating due to reliance on citation metrics, which reflect deeply entrenched biases and exclusionary networks that disadvantage systemically marginalized groups, and these citation metric biases continue to rise globally [20].

Sexism in science publishing is ubiquitous. Women (throughout this manuscript, we use the term “women,” by which we intend to respectfully include and acknowledge the experiences and challenges of all who identify as women and/or womxn and also acknowledge that these and other challenges also exist for nonbinary individuals) are uniformly less cited than men, even though this issue is consistently well acknowledged [21–24]. Recently, the citation gap between genders was found to be as large as 30% across 13 Science, Technology, Engineering, Mathematics, and Medicine (STEMM) disciplines [16], and this gap has been documented across a breadth of journals [25]. These patterns are partially explained by men exhibiting higher rates of self-citation [22,23] and women having shorter career lengths than men [16]. However, decisions on whom to cite may also reflect exclusionary scientific networks that coalesce at scholarly meetings and conferences that, despite recent efforts in improving diversity among participants [26–28], primarily cater to established white men from privileged universities [28–31]. In addition, in comparison to men, women receive more manuscript rejections [32–34], are less likely to be published in prestigious journals (which typically have high citation rates) [35], and are less likely to be invited to write commentaries [36]. These issues may stem from women’s scholarly writing being held to a higher standard than men’s by editors and peer reviewers, placing penalties on women’s productivity, with excessive time spent reworking old research at the cost of conducting new research [37]. Sex-specific differences in manuscript decisions may also arise from conscious and unconscious biases that can impact reviewer assignment [38] and peer review scores [27]. Moreover, the impact of unprofessional peer reviewer comments, defined as “any statement that is unethical or irrelevant to the nature of the work” [39], have disproportionately negative effects for women and nonbinary people relative to men [39]. We also would like to acknowledge that biases experienced by women are likely to be exacerbated for nonbinary individuals, and, so far, little attention has been given to the effects on these groups (but see positive change [40]).

Pervasive racism in science also drives substantial and systemic biases in publication rates, citation rates, and editorial positions [24]. Publication-related metrics show distinct patterns of bias against racially and/or ethnically diverse scientific teams, which experience more than 5% lower acceptance rates and fewer citations than less diverse author teams [41]. Citational segregation—where authors prefer citing authors from the same racial/ethnic group(s)—has been demonstrated with white authors citing other white authors more frequently [24]. This particular bias further reduces the circulation and intellectual acknowledgement of nonwhite scholars’ work and the diversity of viewpoints they bring. Additionally, “high-quality” research is implicitly associated with high-income countries [42], thereby limiting the dissemination of research by scientists from lower-income countries. Moreover, because 98% of scientific journals are published in English, success is related to English proficiency or access to additional editorial support. Scholars who are not fluent in English are at a distinct disadvantage in the publication process, further exacerbating the global gap in citations and research dissemination [43].

Together, gender, racial, and other biases interact and accumulate, often elevating cisgender white males to much higher status than deserved given their contributions to science [44,45]. As such, the unwavering focus on citation-based metrics as indicators of success ignores the breadth of scientific evidence showing these metrics are unreliable, inaccurate, and damaging. While the history, outcome, and current treatment of these biases can vary across disciplines, it is clear that continued use of these metrics perpetuates substantial gender, racial, and ethnic biases, as well as reduced representation of diverse scholarship.

Many efforts to improve diversity in science disciplines have not yet been successful [46–49]. In fact, gender and racial citation biases remain stable or have even worsened over the last half century [16,21–24,41,50], highlighting that efforts to change the system have, by and large, failed to remove systemic biases. Clearly, assessing scientific impact, and thereby assigning value to an individual’s scientific contribution, exclusively—or even primarily—through citations of peer-reviewed literature reflects and amplifies the existing numerous biases that remain embedded within science. Reliance on citation metrics as the primary gauge of impact will continue to limit the advancement of marginalized groups and diminish their scientific contributions [24], representing a loss of diverse talent, perspectives, and approaches.

### (2) Expanding scientific impact beyond citations

Ignoring the breadth of areas where scientists have strong impacts creates an unduly narrow view of the many avenues through which scientists can contribute to intellectual advances, applied science, and equitable communication and translation of science to the public (Fig 1A). This narrow view excludes the real-world impacts within the scientific system (Fig 1B). Even if citation metrics were not biased, using citations as a proxy for success supports the false paradigm that scientists lack impact if they do not (or cannot) publish and/or have chosen “alternative” career paths—a phrase that falsely suggests that academic roles are the only dominant or valued careers for scientists [51,52]. Notably, scholars holding academic positions with high teaching loads, mentoring responsibilities, service requirements, and/or administrative work, as well as those who have chosen careers outside of academia, make critical and diverse contributions to science. Nonacademic careers often place less emphasis on publishing or allow less time to lead or contribute to publications, yet nonetheless provide influential routes for training new scientists, move science broadly into the public realm, and inform critical policy and decision-making [53,54].

Beyond the university classroom and research group, valuing the broader impacts of research is also critical. The cocreation and dissemination of scientific knowledge through collaboration with industry, implementation of government policy, public outreach and media engagement, societal service through science communication, and deferring to the guidance of the communities in which science takes place have the potential to center communities outside research institutions in critical topics [55,56]. Additionally, these intentional actions can aid in restoring public trust in science and promoting the advancement of diverse groups in science careers (e.g., [57]). In fact, funding agencies (e.g., Natural Sciences and Engineering Research Council in Canada, The National Science Foundation in the United States, and the Research Excellence Framework in the United Kingdom) are now including these contributions in the evaluation criteria of the quality of researchers, demonstrating that funding bodies are beginning to play a critical role in normalizing and rewarding the work that scholars do to connect to communities and are key contributors to the valuing of this work. Together, this shift in evaluation criteria indicates that quantifying these impacts is possible and meaningful to science and society more broadly.

Another key avenue of impact on the scientific system is through training upcoming generations of scientists. This role necessitates diverse mentoring and pedagogical skills essential to attract, engage, retain, and elevate scientists in training from different geographies, social–cultural and socioeconomic backgrounds, and career paths. Mentorship is fundamental to efforts to drive a transformation to a fair and safe scientific culture, and we explore the value of mentorship in more detail below in Section 3.

### (3) Broadening the system to value mentorship, diversity, and well-being

Broadening the definitions of success and impact provides an essential foundation to shift the academic system and scientific culture. Through valuing multidimensional mentoring, we can promote decisive actions to improve justice, equity, diversity, and inclusion (Fig 2).

#### Valuing the impact of multidimensional mentorship

Mentorship is a bidirectional relationship that changes as the relationship evolves, and these relationships may vary from being highly formal, structured, and with very specific goals, assignments, and timelines to less formal or clearly articulated relationships [58]. While a traditional mentorship relationship can be between an academic supervisor and a mentee (graduate student, postdoctoral scholar, undergraduate researcher, etc.), mentorship can come in a variety of forms that include peer, supervisor, career development mentor, and/or personal mentor [58,59]. For the purposes of this perspective, we use a broad definition of mentorship that encompasses dynamic mentorship and diverse relationships. We follow the frameworks of scholars who have established the ideas of mentor networks or webs, with mentorship evolving as the needs and aspirations of mentees change through each career stage [60–63].

High-quality mentorship greatly benefits mentees, since mentors are essential in determining career outcomes [64,65]. Research examining a wide range of mentoring relationships (e.g., in government, hospitals, and business) demonstrates that deep engagement in mentorship also leads to a greater sense of job satisfaction, higher commitment to the institution, and higher career success for mentors [66]. Cultivating these outcomes within science could reduce attrition rates often associated with low levels of job satisfaction [67] and a lack of institutional community [68]. The benefits of multidimensional and networked mentoring across career stages, especially by mentors with multiple identities from marginalized groups (i.e., intersectional identities), are critical to increasing representation, recruitment, and retention in the scientific system [58,69–74]. Good mentors can foster a sense of belonging for mentees with diverse backgrounds [75], especially if the mentor belongs to, or strongly associates with, a particular identity, which further emphasizes the importance of inclusive representation in science.

Within academia, outstanding mentorship is invaluable [76,77]. However, this mentorship is traditionally quantified by mentee productivity, which is assessed by the same traditional metrics (e.g., [10]) that have significant biases (see Section 1 above). These metrics fail to acknowledge the diverse value of mentorship, and, thus, reevaluating mentoring practices and how impact is measured will benefit a diverse and intersectional group of early-career scientists [78,79]. We propose that a broader lens of mentorship quality be acknowledged and employed by institutions and funding agencies, which would provide a more holistic measure of scientific impact and reward high-quality mentorship (Figs 1 and 2).

Holistic valuation of mentorship quality includes the contributions from mentors and the achievements of mentees [61,70,80]. In addition to research productivity, metrics encompassing the breadth of mentorship dimensions can incorporate the mentee’s acquired skills, tools provided to the mentee, mentee retention, career commitment, self-efficacy, mentee satisfaction, and overall group culture [81–83]. Mentorship quality could then be quantitatively tracked by institutions throughout an individual’s career within or outside of academia using surveys such as the Global Measure of Mentorship Practices as adapted for STEMM postsecondary education [84]. These metrics could be compared empirically against institutional or national statistics to gauge scientific impact.

Institutions should also elevate strong mentorship by both establishing internal awards for mentor excellence and increasing the weight of such awards in promotions or tenure assessments. Awards such as the National Science Foundation’s Presidential Award for Excellence for Science Math and Engineering Mentoring (PAESMEM), the Australian Museum Eureka Prize, and the Nature Research Awards for Mentoring in Science already exist to recognize outstanding mentors. In addition, placing value on mentorship by funding agencies (e.g., National Science Foundation’s Broader Impacts [85]; Natural Sciences and Engineering Research Council of Canada (NSERC) contributions to training [86]; and Athena Scientific Women’s Academic Network (SWAN) Award Scheme [87]) creates further incentives to achieve mentorship excellence. These prestigious recognitions, coupled with funding and incentives to support mentees from marginalized groups, represent strong steps forward in valuing mentoring and highlighting the efforts and impacts of individuals working to support the next generation of researchers from diverse backgrounds and/or identities.

#### Mentorship can promote justice, equity, diversity, and inclusion in science

An important avenue in promoting justice, equity, diversity, and inclusion in science is through effective mentorship strategies beyond the traditional dyadic and top-down relationships, such as creating mentorship networks as we discussed above [60,88]. The idea of a comprehensive, singular mentor suggests that one person can meet all mentee needs; however, each individual has unique needs—especially those who identify as members of systemically marginalized groups [89–91]. For example, some individuals may seek mentorship for academic and career advice alone, while others may pursue mentors with similar personal identities or experiences for support with the unique situations these individuals face in science. Mentoring networks are effective in allowing individuals to seek out appropriate mentorships to meet their identified goals and associated needs. By implementing the use of mentorship networks for researchers at all levels, institutions can better connect individuals with appropriate mentors that can support the success of one another [60,90]. Because mentorship networks support the long-term career goals of the mentees [92], institutions that support these networks are investing in higher retention of scientists from marginalized groups [93].

Mentoring should not be limited to students or early career stages. Mentoring throughout one’s career provides important mechanisms for learning new skills, broadening career path options, and attaining life goals. For example, formal mentoring could be available for individuals transitioning toward administration. This is particularly important for helping to build a more equitable community as administrators often hold the most power for implementing change at institutions. If such leaders are trained in how to build inclusive cultures, change will likely be quicker and broader.

While multidimensional mentoring will facilitate a more inclusive culture, specific strategies are also needed to change the systemic sexism and racism that pervade institutions [93,94]. A first step is to identify barriers to diversity, followed by policies and training designed to support transformative institutional change [93]. These include shifting community culture through communication, collaboration, and training to support interventions and leadership. For example, transitioning from a “gatekeeping” to a “groundskeeping” approach at all levels of the academic hierarchy is a key component of the required shift in culture to address pervasive obstacles to justice, equity, diversity, and inclusion [95].

For these efforts to be achieved, faculty and researchers need to be educated and supported with structured programs that embed these principles in teaching, research, and mentoring (e.g., [96]). For example, training in inclusive pedagogical approaches (i.e., inclusive or deep teaching [97,98]), bystander intervention training, and anti-bullying and antiracist mentoring and teaching practices can be made part of the job expectation for those in supervisory and mentoring roles. This training may also include critical pedagogy that examines and challenges the systems of oppression that shape society [99–101] and promotes both the intellectual growth and well-being of students and mentees [102]. To ensure that training opportunities become valued by participants, institutions may consider implementing mandatory participation by requiring training for career advancement or as prerequisites for recruiting mentees. However, training programs should be mindfully designed to engage those who may complete training for inauthentic reasons. Discussions of topics covered in training should become standard practice at regular events including faculty meetings and retreats and graduate student association meetings. Undergraduate programs can include discussions of unconscious bias and how such biases influence classroom dynamics. When it comes to hiring, candidates should be assessed across multiple axes to ensure the recruitment of individuals who are dedicated to building a stronger and more equitable community. Leaders need to work hard to develop creative ways to promote justice, equity, diversity, and inclusion in science to dismantle the barriers that prevent healthy and innovative science workplaces. We therefore advocate for the continuation and development of awards and incentives that recognize and reward authentic efforts to do so.

Unfortunately, large gaps in the implementation of effective strategies to dismantle discriminatory systems still persist. Over the last decade, a range of initiatives in academia, industry, and government have been implemented to support the attraction, retention, and progression of people from systemically marginalized groups at national and international levels. To normalize and move these initiatives forward, we must leverage the many recommendations that have already been made for justice, equity, diversity, and inclusion in science [5–7,96,103,104]. Evaluating these actions and policies within a scientific framework and developing best practices is a start to implementing effective strategies [105]. Scientific institutions and funding agencies must implement initiatives that address the systemic discrimination and biases in student admissions, recruitment, grant funding, and promotions [93,106–109]. Institutional commitment is needed to strategically implement meaningful equity and inclusion approaches with effective accountability mechanisms in place [110].

#### Transformed science culture: Supporting a safe and healthy environment

The role of inclusive mentoring practices (e.g., sponsoring, counseling, networking, and advocating; Fig 2) is unequivocal in providing essential tools to foster justice, equity, diversity, and inclusion for mentees, preventing toxic mentor–mentee relationships, and overcoming barriers and access in STEMM careers [111,112]. Social belonging and valuing of multiple identities in science reinforces achievement [9,96,113,114], and diverse teams have been shown to increase the rate of innovation and collective creativity [115–117]. While good mentorship can foster a sense of belonging in science for the mentee, relationships of many mentees from marginalized groups with their mentors—who are often from the majority group—are not always positive, leading to health issues, such as insomnia and anxiety [118], and lower retention of these groups in science (reviewed in [93,104]). In order to effectively mentor, all mentors—particularly those who are not familiar with the experiences and perspectives of systemically marginalized scholars—should engage with cultures, communities, and perspectives that differ from their own, connect with communities that are working toward creating justice, equity, diversity, and inclusion, and support institutional change already underway. In addition, increasing representation from marginalized communities throughout institutional hierarchies provides greater opportunities for mentees to find mentors with which to build meaningful relationships.

Of particular concern is the recently highlighted decline in mental health of many academics and a growing crisis at the graduate level [119]. Graduate students are at least twice as likely to experience mental health challenges, such as anxiety and depression, compared to the general population with equivalent education [120]. This trend is even more striking for women of color in STEMM, who are facing systemic sexism and racism, along with daily microaggressions and safety concerns [121]. Sexual minorities and LGBTQ+-identifying people are also subject to discrimination that adversely affects their well-being, mental health and, ultimately, retention in STEMM fields [74,104]. Laboratory work, field work, and simple existence in the academy can often place marginalized groups, including those with disabilities, at risk of injury, harassment, bullying, and assault (e.g., [103,122–124]). To combat these challenges, specific strategies for safety and well-being [125,126] must be supported at the research group, departmental, institutional, and funding organization levels.

Moreover, destructive mentoring has frequently gone unchecked in academia [123], often because of the appearance of a superficially productive, well-functioning, or supportive working environment. This is in large part due to power dynamics within the formal mentor–mentee relationship, as academia was constructed on a model with a top-down hierarchy (Fig 2). Key future directions to redress this issue include proactive policies at the institutional and departmental levels, which could include formalizing mentee and advisor responsibilities and expectations [127,128]. Initiatives can be tailored to empower mentees to manage their relationship with their research mentors and for faculty to advise, educate, and supervise using inclusive techniques [112]. Further, there should be clear procedures to change behaviors displayed by potentially abusive mentors and significant consequences to ensure the prevention of negative impacts on future mentees (e.g., [129]). Actions such as facilitating safe ways in which mentees can provide feedback to their mentor—whether positive or negative—is a start to empowering mentees and aligning expectations [130]. Institutional oversight in developing a strong mentorship culture, support for mentor–mentee training, and responsibility for administrative interventions are critical aspects of ensuring a safe environment for all.

Institutions are at the foundation of creating a culture that promotes community wellness, beginning with a clear mission that aligns with the safety and health of mentees and mentors, especially those from marginalized groups [131]. Indeed, it is the institution’s responsibility to ensure there is a specific training focused on effective mentoring practices and modeling wellness for mentees [93,132]. Mentees and mentors need to be trained to appropriately flag, assess, and address mental health and safety concerns using targeted and early-intervention roadmaps in safe spaces. This training should be made readily available via a variety of modalities, such as mental health first aid training (e.g., [133]). An enhanced focus on health, safety, and accessibility in science, as well as institutional support for mentorship assessment and growth, will lead to improved retention of scientists from diverse backgrounds and increased community health and wellness (Fig 2). While it has become increasingly standard for institutions to publicly profess commitments to justice, equity, diversity, and inclusion, without sufficient investment of time, energy, and funding, these commitments will remain performative [134].

## Conclusions

To dismantle the problematic reward system and create an inclusive and innovative community, the scientific system needs to move beyond the current narrow measures of success and impact to focus on holistic assessment (Fig 1). Acknowledging that there is a diverse range of contributions and career pathways will broaden the value system in science. By embracing inclusive approaches and not forcing people to assimilate into sexist and racist norms, we can grow a more equitable model for science that addresses and actively works to counter injustices. The challenges associated with changing a deeply embedded institutional history, culture, and structure toward a different inclusive value system will require institutions to champion a “new norm” to bring change at a global scale. Such a shift must be embraced by all and led by those currently in positions of power and privilege. This shift requires not only specific proactive actions and reforms to institutionalize change but also mechanisms to monitor implementation and provide feedback optimizing an adaptive and dynamic structure.

## Abbreviations

LGBTQ+: lesbian, gay, bisexual, transgender, queer, and others
NSERC: Natural Sciences and Engineering Research Council of Canada
PAESMEM: Presidential Awards for Excellence in Science, Mathematics and Engineering Mentoring
STEMM: Science, Technology, Engineering, Mathematics, and Medicine
SWAN: Scientific Women’s Academic Network

## References

[1] Henley MM. Women’s Success in Academic Science: Challenges to Breaking Through the Ivory Ceiling. Sociol Compass. 2015;9:668–80.

[2] Courchamp F, Bradshaw CJA. 100 articles every ecologist should read. Nat Ecol Evol. 2018;2:395–401. doi: 10.1038/s41559-017-0370-9 29133900

[3] Sterling AD, Thompson ME, Wang S, Kusimo A, Gilmartin S, Sheppard S. The confidence gap predicts the gender pay gap among STEM graduates. Proc Natl Acad Sci U S A. 2020;117:30303–8. doi: 10.1073/pnas.2010269117 33199594 PMC7720106

[4] AlShebli B, Makovi K, Rahwan T. The association between early career informal mentorship in academic collaborations and junior author performance. Nat Commun. 2020;11:5855. doi: 10.1038/s41467-020-19723-8 33203848 PMC7672107

[5] Greider CW, Sheltzer JM, Cantalupo NC, Copeland WB, Dasgupta N, Hopkins N, et al. Increasing gender diversity in the STEM research workforce. Science. 2019;366:692–5. doi: 10.1126/science.aaz0649 31699926

[6] Barber PH, Hayes TB, Johnson TL, Márquez-Magaña L. 10,234 signatories Systemic racism in higher education. Science. 2020;369:1440–1. doi: 10.1126/science.abd7140 32943517

[7] Chaudhary VB, Berhe AA. Ten simple rules for building an antiracist lab. PLoS Comput Biol. 2020;16:e1008210. doi: 10.1371/journal.pcbi.1008210 33001989 PMC7529192

[8] Smith JL, Handley IM, Zale AV, Rushing S, Potvin MA. Now Hiring! Empirically Testing a Three-Step Intervention to Increase Faculty Gender Diversity in STEM. Bioscience. 2015;65:1084–7. doi: 10.1093/biosci/biv138 26955075 PMC4777060

[9] Allen-Ramdial S-AA, Campbell AG. Reimagining the Pipeline: Advancing STEM Diversity, Persistence, and Success. Bioscience. 2014;64:612–8. doi: 10.1093/biosci/biu076 25561747 PMC4282132

[10] Ma Y, Mukherjee S, Uzzi B. Mentorship and protégé success in STEM fields. Proc Natl Acad Sci U S A. 2020;117:14077–83. doi: 10.1073/pnas.1915516117 32522881 PMC7322065

[11] Way SF, Morgan AC, Larremore DB, Clauset A. Productivity, prominence, and the effects of academic environment. Proc Natl Acad Sci U S A. 2019;116:10729–33. doi: 10.1073/pnas.1817431116 31036658 PMC6561156

[12] Crane D. Scientists at major and minor universities: a study of productivity and recognition. Am Sociol Rev. 1965;30:699–714. 5824935

[13] Deutsch FM, Yao B. Gender differences in faculty attrition in the USA. Community Work Fam. 2014:392–408. doi: 10.1080/13668803.2014.885880

[14] Xu YJ. Gender Disparity in STEM Disciplines: A Study of Faculty Attrition and Turnover Intentions. Res High Educ. 2008:607–24. doi: 10.1007/s11162-008-9097-4

[15] Bennett CL, Salinas RY, Locascio JJ, Boyer EW. Two decades of little change: An analysis of U.S. medical school basic science faculty by sex, race/ethnicity, and academic rank. PLoS ONE. 2020:e0235190. doi: 10.1371/journal.pone.0235190 32735593 PMC7394429

[16] Huang J, Gates AJ, Sinatra R, Barabási A-L. Historical comparison of gender inequality in scientific careers across countries and disciplines. Proc Natl Acad Sci U S A. 2020;117:4609–16. doi: 10.1073/pnas.1914221117 32071248 PMC7060730

[17] Box-Steffensmeier JM, Cunha RC, Varbanov RA, Hoh YS, Knisley ML, Holmes MA. Survival Analysis of Faculty Retention and Promotion in the Social Sciences by Gender. PLoS ONE. 2015;10:e0143093. doi: 10.1371/journal.pone.0143093 26580565 PMC4651362

[18] Khan MS, Lakha F, Tan MMJ, Singh SR, Quek RYC, Han E, et al. More talk than action: gender and ethnic diversity in leading public health universities. Lancet. 2019;393:594–600. doi: 10.1016/S0140-6736(18)32609-6 30739695

[19] Clauset A, Arbesman S, Larremore DB. Systematic inequality and hierarchy in faculty hiring networks. Sci Adv. 2015;1:e1400005. doi: 10.1126/sciadv.1400005 26601125 PMC4644075

[20] Nielsen MW, Andersen JP. Global citation inequality is on the rise. Proc Natl Acad Sci U S A. 2021;118. doi: 10.1073/pnas.2012208118 33558230 PMC7896328

[21] Caplar N, Tacchella S, Birrer S. Quantitative evaluation of gender bias in astronomical publications from citation counts. Nat Astron. 2017;1:0141.

[22] King MM, Bergstrom CT, Correll SJ, Jacquet J, West JD. Men Set Their Own Cites High: Gender and Self-citation across Fields and over Time. Soc Forces. 2017;3:2378023117738903.

[23] Dworkin JD, Linn KA, Teich EG, Zurn P, Shinohara RT, Bassett DS. The extent and drivers of gender imbalance in neuroscience reference lists. Nat Neurosci. 2020;23:918–26. doi: 10.1038/s41593-020-0658-y 32561883

[24] Chakravartty P, Kuo R, Grubbs V, C. #CommunicationSoWhite. J Commun. 2018:254–66. doi: 10.1093/joc/jqy003

[25] Bendels MHK, Müller R, Brueggmann D, Groneberg DA. Gender disparities in high-quality research revealed by Nature Index journals. PLoS ONE. 2018;13:e0189136. doi: 10.1371/journal.pone.0189136 29293499 PMC5749692

[26] Burnett NP, King EE, Salcedo MK, Tanner RL, Wilsterman K. Conference scheduling undermines diversity efforts. Nat Ecol Evol. 2020;4:1283–4. doi: 10.1038/s41559-020-1276-5 32747776

[27] Johnson CY, Chin. Improving Diversity and Promoting Inclusion in the Society for Epidemiologic Research Through Choice of Conference Location. Am J Epidemiol. 2020;189:1030–2. doi: 10.1093/aje/kwaa107 32602521 PMC7666409

[28] Fleming N. How to organize a conference that’s open to everyone. Nature. 2019;571:S46–7. doi: 10.1038/d41586-019-02253-9 31341294

[29] Witze A. How to counter “manels” and make scientific meetings more inclusive. Nature. 2019. doi: 10.1038/d41586-019-01022-y 32235919

[30] King L, MacKenzie L, Tadaki M, Cannon S, McFarlane K, Reid D, et al. Diversity in geoscience: Participation, behaviour, and the division of scientific labour at a Canadian geoscience conference. Facets (Ott). 2018;3:415–40.

[31] Shishkova E, Kwiecien NW, Hebert AS, et al. Gender diversity in a STEM subfield–analysis of a large scientific society and its annual conferences. J Am Soc Mass Spectrom. 2017. Available from: https://pubs.acs.org/doi/abs/10.1021/jasms.8b05451?casa_tokenGU-dhjf8H34AAAAA:2r3UnqFWAVvt4A_ejayXzuVYUKaBEcaOLEATA8dJJVMe-XeHel9Lxcnx57qNUU0W_zr9VUQB-nQbNyg3. 10.1007/s13361-017-1803-z PMC5856480 28952050

[32] Hagan AK, Topçuoğlu BD, Gregory ME, Barton HA, Schloss PD. Women Are Underrepresented and Receive Differential Outcomes at ASM Journals: a Six-Year Retrospective Analysis. MBio. 2020;11. doi: 10.1128/mBio.01680-20 33262256 PMC7733940

[33] Fox CW, Paine CET. Gender differences in peer review outcomes and manuscript impact at six journals of ecology and evolution. Ecol Evol. 2019;9:3599–619. doi: 10.1002/ece3.4993 30962913 PMC6434606

[34] Niriella MA, De Silva AP, de Silva HJ, Jayasinghe S. Is there racism in academic medical publishing? BMJ Evid Based Med. 2020. doi: 10.1136/bmjebm-2020-111487 32723765

[35] Holman L, Stuart-Fox D, Hauser CE. The gender gap in science: How long until women are equally represented? PLoS Biol. 2018;16:e2004956. doi: 10.1371/journal.pbio.2004956 29672508 PMC5908072

[36] Thomas EG, Jayabalasingham B, Collins T, Geertzen J, Bui C, Dominici F. Gender Disparities in Invited Commentary Authorship in 2459 Medical Journals. JAMA Netw Open. 2019;2:e1913682. doi: 10.1001/jamanetworkopen.2019.13682 31642926 PMC6820037

[37] Hengel E. Evidence from peer review that women are held to higher standards. 2017. https://livrepository.liverpool.ac.uk/3018341/1/voxeu.pdf.

[38] Helmer M, Schottdorf M, Neef A, Battaglia D. Gender bias in scholarly peer review. Elife. 2017;6. doi: 10.7554/eLife.21718 28322725 PMC5360442

[39] Silbiger NJ, Stubler AD. Unprofessional peer reviews disproportionately harm underrepresented groups in STEM. PeerJ. 2019:e8247. doi: 10.7717/peerj.8247 31844596 PMC6911688

[40] Gaskins LC, McClain CR. Visible name changes promote inequity for transgender researchers. PLoS Biol. 2021;19:e3001104. doi: 10.1371/journal.pbio.3001104 33690606 PMC7943012

[41] Lerback JC, Hanson B, Wooden P. Association Between Author Diversity and Acceptance Rates and Citations in Peer-Reviewed Earth Science Manuscripts. Earth Space Sci. 2020. doi: 10.1029/2019ea000946

[42] Harris M, Macinko J, Jimenez G, Mullachery P. Measuring the bias against low-income country research: an Implicit Association Test. Glob Health. 2017;13:80. 10.1186/s12992-017-0304-y PMC5674740 29110668

[43] Ramírez-Castañeda V. Disadvantages in preparing and publishing scientific papers caused by the dominance of the English language in science: The case of Colombian researchers in biological sciences. PLoS ONE. 2020;15:e0238372. doi: 10.1371/journal.pone.0238372 32936821 PMC7494110

[44] Lincoln AE, Pincus S, Koster JB, Leboy PS. The matilda effect in science: awards and prizes in the US, 1990s and 2000s. Soc Stud Sci. 2012;42:307–20. doi: 10.1177/0306312711435830 22849001

[45] Rossiter MW. The Matthew Matilda Effect in Science. Soc Stud Sci. 1993:325–41. doi: 10.1177/030631293023002004

[46] Dutt K. Race and racism in the geosciences. Nat Geosci. 2020:2–3. doi: 10.1038/s41561-019-0519-z

[47] D’Armiento J, Witte SS, Dutt K, Wall M, McAllister G. Achieving women’s equity in academic medicine: challenging the standards. Lancet. 2019:e15–6. doi: 10.1016/S0140-6736(19)30234-X 30739701

[48] Bernard RE, Cooperdock EHG. No progress on diversity in 40 years. Nat Geosci. 2018:292–5. doi: 10.1038/s41561-018-0116-6

[49] Puritty C, Strickland LR, Alia E, Blonder B, Klein E, Kohl MT, et al. Without inclusion, diversity initiatives may not be enough. Science. 2017:1101–2. doi: 10.1126/science.aai9054 28912234

[50] Andersen JP, Schneider JW, Jagsi R, Nielsen MW. Gender variations in citation distributions in medicine are very small and due to self-citation and journal prestige. Elife. 2019;8. doi: 10.7554/eLife.45374 31305239 PMC6677534

[51] Sauermann H, Roach M. Science PhD career preferences: levels, changes and advisor encouragement. PLoS ONE. 2012;7:e36307. doi: 10.1371/journal.pone.0036307 22567149 PMC3342243

[52] Roach M, Sauermann H. The declining interest in an academic career. PLoS ONE. 2017;12:e0184130. doi: 10.1371/journal.pone.0184130 28922403 PMC5602526

[53] Pham D. Public engagement is key for the future of science research. NPJ Sci Learn. 2016;1:16010. doi: 10.1038/npjscilearn.2016.10 30792895 PMC6380378

[54] Funk C, Rainie L, Page D. Public and scientists’ views on science and society. Pew Research Center. 2015;29.

[55] van der Linden S, Maibach E, Leiserowitz A. Improving Public Engagement With Climate Change: Five “Best Practice” Insights From Psychological Science. Perspect Psychol Sci. 2015;10:758–63. doi: 10.1177/1745691615598516 26581732

[56] Plohl N, Musil B. Modeling compliance with COVID-19 prevention guidelines: the critical role of trust in science. Psychol Health Med. 2021;26:1–12. doi: 10.1080/13548506.2020.1772988 32479113

[57] Henderson RI, Williams K, Crowshoe LL. Mini-med school for Aboriginal youth: experiential science outreach to tackle systemic barriers. Med Educ Online. 2015;20:29561. doi: 10.3402/meo.v20.29561 26701840 PMC4689949

[58] Hernandez PR, Bloodhart B, Barnes RT, Adams AS, Clinton SM, Pollack I, et al. Promoting professional identity, motivation, and persistence: Benefits of an informal mentoring program for female undergraduate students. PLoS ONE. 2017;12:e0187531. doi: 10.1371/journal.pone.0187531 29091969 PMC5665547

[59] Grant CS. Mentoring. Success Strategies From Women in STEM. 2015:63–96.

[60] Montgomery BL. Mapping a Mentoring Roadmap and Developing a Supportive Network for Strategic Career Advancement. SAGE Open. 2017;7:2158244017710288.

[61] Montgomery BL, Dodson JE, Johnson SM. Guiding the Way: Mentoring Graduate Students and Junior Faculty for Sustainable Academic Careers. SAGE Open. 2014;4:2158244014558043.

[62] Essay calling for senior faculty to embrace new style of mentoring. [cited 2021 Apr 16]. https://www.insidehighered.com/advice/2013/07/22/essay-calling-senior-faculty-embrace-new-style-mentoring.

[63] When it comes to mentoring, the more the merrier. [cited 2021 Apr 16]. https://community.chronicle.com/news/326-when-it-comes-to-mentoring-the-more-the-merrier.

[64] Herrmann SD, Adelman RM, Bodford JE, Graudejus O, Okun MA, Kwan VSY. The Effects of a Female Role Model on Academic Performance and Persistence of Women in STEM Courses. Basic Appl Soc Psychol. 2016;38:258–68.

[65] Dasgupta N, Scircle MM, Hunsinger M. Female peers in small work groups enhance women’s motivation, verbal participation, and career aspirations in engineering. Proc Natl Acad Sci U S A. 2015;112:4988–93. doi: 10.1073/pnas.1422822112 25848061 PMC4413283

[66] Ghosh R, Reio TG. Career benefits associated with mentoring for mentors: A meta-analysis. J Vocat Behav. 2013:106–16. doi: 10.1016/j.jvb.2013.03.011

[67] Misra J, Lundquist JH, Templer. Gender, Work Time, and Care Responsibilities Among Faculty1. Sociol Forum. 2012:300–23. doi: 10.1111/j.1573-7861.2012.01319.x

[68] Denson N, Szelényi K, Bresonis K. Correlates of Work-Life Balance for Faculty Across Racial/Ethnic Groups. Res High Educ. 2018:226–47. doi: 10.1007/s11162-017-9464-0

[69] Hernandez PR, Bloodhart B, Adams AS, Barnes RT, Burt M, Clinton SM, et al. Role modeling is a viable retention strategy for undergraduate women in the geosciences. Geosphere. 2018;14:2585–93.

[70] Hernandez PR, Adams AS, Barnes RT, Bloodhart B, Burt M, Clinton SM, et al. Inspiration, inoculation, and introductions are all critical to successful mentorship for undergraduate women pursuing geoscience careers. Commun Earth Environ. 2020;1:1–9.

[71] Dennehy TC, Dasgupta N. Female peer mentors early in college increase women’s positive academic experiences and retention in engineering. Proc Natl Acad Sci U S A. 2017;114:5964–9. doi: 10.1073/pnas.1613117114 28533360 PMC5468611

[72] Barnes BJ, Williams EA, Stassen MLA. Dissecting doctoral advising: a comparison of students’ experiences across disciplines. J Furth High Educ. 2012:309–31. doi: 10.1080/0309877x.2011.614933

[73] Hund AK, Churchill AC, Faist AM, Havrilla CA, Love Stowell SM, McCreery HF, et al. Transforming mentorship in STEM by training scientists to be better leaders. Ecol Evol. 2018;8:9962–74. doi: 10.1002/ece3.4527 30397439 PMC6206201

[74] Hughes BE. Coming out in STEM: Factors affecting retention of sexual minority STEM students. Sci Adv. 2018;4:eaao6373. doi: 10.1126/sciadv.aao6373 29546240 PMC5851677

[75] O’Brien LT, Bart HL, Garcia DM. Why are there so few ethnic minorities in ecology and evolutionary biology? Challenges to inclusion and the role of sense of belonging. Soc Psychol Educ. 2020;23:449–77.

[76] Downing RA, Crosby FJ, Blake-Beard S. The Perceived Importance of Developmental Relationships on Women Undergraduates’ Pursuit of Science. Psychol Women Q. 2005;29:419–26.

[77] Fried T, MacCleave A. Influence of role models and mentors on female graduate students’ choice of science as a career. Alberta J Educ Res. 2009;55:482–96.

[78] Knepper HJ, Scutelnicu G, Tekula R. Why gender and research productivity matters in academia: Exploring evidence from NASPAA-accredited schools. J Public Aff Educ. 2020:51–72. doi: 10.1080/15236803.2019.1565066

[79] Garmire LX. Mentorship is not co-authorship: a revisit to mentorship. Genome Biol. 2021;22:1–3. doi: 10.1186/s13059-020-02207-9 33397414 PMC7780662

[80] Montgomery BL. From deficits to possibilites: Mentoring lessons from plants on cultivating individual growth through environmental assessment and optimization. Public Philos J. 2018.

[81] Paglis LL, Green SG, Bauer TN. Does adviser mentoring add value? A longitudinal study of mentoring and doctoral student outcomes. Res High Educ. 2006;47:451–76.

[82] Sambunjak D, Straus SE, Marusić A. Mentoring in academic medicine: a systematic review. JAMA. 2006;296:1103–15. doi: 10.1001/jama.296.9.1103 16954490

[83] Thomas N, Bystydzienski J, Desai A. Changing Institutional Culture through Peer Mentoring of Women STEM Faculty. Innov High Educ. 2015;40:143–57.

[84] Tenenbaum HR, Crosby FJ, Gliner MD. Mentoring Relationships in Graduate School. J Vocat Behav. 2001:326–41. doi: 10.1006/jvbe.2001.1804

[85] US NSF—: Broader impacts review criterion—dear colleague letter nsf07046. [cited 2021 Jan 23]. https://www.nsf.gov/pubs/2007/nsf07046/nsf07046.jsp.

[86] Government of Canada, Natural Sciences, Engineering Research Council of Canada, Communications Division. NSERC—Policy and Guidelines on Contributions to Research and Training. 2016 [cited 2021 Jan 23]. https://www.nserc-crsng.gc.ca/NSERC-CRSNG/Policies-Politiques/assesscontrib-evalcontrib_eng.asp.

[87] Kalpazidou Schmidt E, Ovseiko PV, Henderson LR, Kiparoglou V. Understanding the Athena SWAN award scheme for gender equality as a complex social intervention in a complex system: analysis of Silver award action plans in a comparative European perspective. Health Res Policy Syst. 2020;18:19. doi: 10.1186/s12961-020-0527-x 32059678 PMC7023775

[88] National Academies of Sciences, Engineering, and Medicine; Policy and Global Affairs; Board on Higher Education and Workforce; Committee on Effective Mentoring, Dahlberg ML, Byars-Winston A. Summary. National Academies Press (US); 2019.

[89] Bertrand Jones T, Ford JR, Pierre DF, Davis-Maye D. Thriving in the Academy: Culturally Responsive Mentoring for Black Women’s Early Career Success. In: Crimmins G, editor. Strategies for Supporting Inclusion and Diversity in the Academy: Higher Education, Aspiration and Inequality. Cham: Springer International Publishing; 2020. pp. 123–140.

[90] Yun JH, Baldi B, Sorcinelli MD. Mutual mentoring for early-career and underrepresented faculty: Model, research and practice. Innov High Educ. 2016;41:441–51.

[91] Essay on mentoring and minority faculty members. [cited 2021 Apr 16]. https://www.insidehighered.com/advice/2011/11/14/essay-mentoring-and-minority-faculty-members.

[92] Higgins MC, Constellations TDA. Careers: Toward Understanding the Effects of Multiple Developmental Relationships. J Organ Behav. 2001;22:223–47.

[93] Whittaker J. A., Montgomery B. L., Martinez Acosta V. G. Eds. Retention of Underrepresented Minority Faculty: Strategic Initiatives for Institutional Value Proposition Based on Perspectives from a Range of Academic Institutions. J Undergrad Neurosci Educ. 2015;13:A136–45. 26240521 PMC4521729

[94] August L, Culture WJ. Climate, and Contribution: Career Satisfaction Among Female Faculty. Res High Educ. 2004:177–92. doi: 10.1023/b:rihe.0000015694.14358.ed

[95] Montgomery BL. Academic Leadership: Gatekeeping or Groundskeeping? JVBL. 2020;13:16.

[96] Núñez A-M, Rivera J, Hallmark T. Applying an intersectionality lens to expand equity in the geosciences. J Geosci Educ. 2020:97–114. doi: 10.1080/10899995.2019.1675131

[97] Dewsbury BM. Deep teaching in a college STEM classroom. Cult Stud Sci Educ. 2020;15:169–91.

[98] Dewsbury BM, Brame. Inclusive Teaching. CBE Life Sci Educ. 2019;18:fe2. doi: 10.1187/cbe.19-01-0021 31025917 PMC7058128

[99] Patton LD. Disrupting Postsecondary Prose: Toward a Critical Race Theory of Higher Education. Urban Educ. 2016;51:315–42.

[100] Haynes C, Patton LD. From Racial Resistance to Racial Consciousness: Engaging White STEM Faculty in Pedagogical Transformation. J Cases Educ Leadersh. 2019;22:85–98.

[101] Freire P. Pedagogy of the Oppressed: 50th Anniversary Edition. Bloomsbury Publishing USA; 2018.

[102] Hooks B. Teaching To Transgress. Routledge. 1994.

[103] Clancy KBH, Cortina LM, Kirkland. Opinion: Use science to stop sexual harassment in higher education. Proc Natl Acad Sci U S A. 2020;117:22614–8. doi: 10.1073/pnas.2016164117 32817430 PMC7502731

[104] Freeman JB. Measuring and Resolving LGBTQ Disparities in STEM. Policy Insights Behav Brain Sci. 2020;7:141–8.

[105] McKinnon M. The absence of evidence of the effectiveness of Australian gender equity in STEM initiatives. Aust J Soc Issues. 2020;77:542.

[106] Taffe MA, Gilpin NW. Equity, Diversity and Inclusion: Racial inequity in grant funding from the US National Institutes of Health. Elife. 2021;10:e65697. doi: 10.7554/eLife.65697 33459595 PMC7840175

[107] Stevens KR, Masters KS, Imoukhuede PI, Haynes KA, Setton LA, Cosgriff-Hernandez E, et al. Fund Black scientists. Cell. 2021;184:561–5. doi: 10.1016/j.cell.2021.01.011 33503447

[108] Kaatz A, Lee Y-G, Potvien A, Magua W, Filut A, Bhattacharya A, et al. Analysis of National Institutes of Health R01 Application Critiques, Impact, and Criteria Scores: Does the Sex of the Principal Investigator Make a Difference? Acad Med. 2016;91:1080–8. doi: 10.1097/ACM.0000000000001272 27276003 PMC4965296

[109] Bhalla N. Strategies to improve equity in faculty hiring. Mol Biol Cell. 2019;30:2744–9. doi: 10.1091/mbc.E19-08-0476 31609672 PMC6789160

[110] Mangubhai S, Lawless S. Exploring gender inclusion in small-scale fisheries management and development in Melanesia. Mar Policy. 2021;123:104287.

[111] Kendricks KD, Nedunuri KV, Arment AR. Minority student perceptions of the impact of mentoring to enhance academic performance in STEM disciplines. J STEM Educ. 2013;14. Available from: https://www.researchgate.net/profile/Krishnakumar_Nedunuri/publication/332857816_Minority_Student_Perceptions_of_the_Impact_of_Mentoring_to_Enhance_Academic_Performance_in_STEM_Disciplines/links/5cccf3d0458515712e9033f0/Minority-Student-Perceptions-of-the-Impact-of-Mentoring-to-Enhance-Academic-Performance-in-STEM-Disciplines.pdf.

[112] Clement L, Leung KN, Lewis JB, Saul NM. The Supervisory Role of Life Science Research Faculty: The Missing Link to Diversifying the Academic Workforce? J Microbiol Biol Educ. 2020;21. doi: 10.1128/jmbe.v21i1.1911 32341732 PMC7173632

[113] Cohen GL, Identity GJ. Belonging, and Achievement: A Model, Interventions, Implications. Curr Dir Psychol Sci. 2008;17:365–9.

[114] Wilson D, Jones D, Bocell F, Crawford J, Kim MJ, Veilleux N, et al. Belonging and academic engagement among undergraduate STEM students: A multi-institutional study. Res High Educ. 2015;56:750–76.

[115] Hofstra B, Kulkarni VV, Munoz-Najar Galvez S, He B, Jurafsky D, McFarland DA. The Diversity-Innovation Paradox in Science. Proc Natl Acad Sci U S A. 2020;117:9284–91. doi: 10.1073/pnas.1915378117 32291335 PMC7196824

[116] Bassett-Jones N. The Paradox of Diversity Management, Creativity and Innovation. Creat Innov Manag. 2005;14:169–75.

[117] Woolley A, Malone T. What makes a team smarter? More women. Harv Bus Rev. 2011;89:32–3. 21714385

[118] Al Makhamreh M, Stockley D. Mentorship and well-being: Examining doctoral students’ lived experiences in doctoral supervision context. Int J Mentor Coach Educ. 2019;39:88.

[119] Evans TM, Bira L, Gastelum JB, Weiss LT, Vanderford NL. Evidence for a mental health crisis in graduate education. Nat Biotechnol. 2018;36:282–4. doi: 10.1038/nbt.4089 29509732

[120] Levecque K, Anseel F, De Beuckelaer A, Van der Heyden J, Gisle L. Work organization and mental health problems in PhD students. Res Policy. 2017;46:868–879.

[121] Wilkins-Yel KG, Hyman J, Zounlome NOO. Linking intersectional invisibility and hypervisibility to experiences of microaggressions among graduate women of color in STEM. J Vocat Behav. 2019:51–61. doi: 10.1016/j.jvb.2018.10.018

[122] Anadu J, Ali H, Jackson C. Ten steps to protect BIPOC scholars in the field. Eos. 2020;101. doi: 10.1029/2020eo150525

[123] National Academies of Sciences, Engineering, and Medicine, Policy and Global Affairs, Committee on Women in Science, Engineering, and Medicine, Committee on the Impacts of Sexual Harassment in Academia. Sexual Harassment of Women: Climate, Culture, and Consequences in Academic Sciences, Engineering, and Medicine. National Academies Press; 2018. 29894119

[124] St. John K, Riggs E, Mogk D. Sexual Harassment in the Sciences: A Call to Geoscience Faculty and Researchers to Respond. J Geosci Educ. 2016;64:255–257.

[125] Demery A-JC, Pipkin MA. Safe fieldwork strategies for at-risk individuals, their supervisors and institutions. Nat Ecol Evol. 2021;5:5–9. doi: 10.1038/s41559-020-01328-5 33046873

[126] Morales N, Bisbee O’Connell K, McNulty S, Berkowitz A, Bowser G, Giamellaro M, et al. Promoting inclusion in ecological field experiences: Examining and overcoming barriers to a professional rite of passage. Bull Ecol Soc Am. 2020;101. doi: 10.1002/bes2.1742

[127] Masters KS, Kreeger PK. Ten simple rules for developing a mentor–mentee expectations document. PLoS Comput Biol. 2017;13:e1005709. doi: 10.1371/journal.pcbi.1005709 28934208 PMC5608163

[128] Omar F, Mahone JP, Ngobia J, FitzSimons J. Building Rapport Between International Graduate Students and Their Faculty Advisors: Cross-Cultural Mentoring Relationships at the University of Guelph. CJSoTL. 2016;7. doi: 10.5206/cjsotl-rcacea.2016.2.8

[129] Ramani S, Gruppen L, Kachur EK. Twelve tips for developing effective mentors. Med Teach. 2006;28. doi: 10.1080/01421590600825326 16973451

[130] Anderson L, Silet K, Fleming M. Evaluating and giving feedback to mentors: new evidence-based approaches. Clin Transl Sci. 2012;5:71–7. doi: 10.1111/j.1752-8062.2011.00361.x 22376261 PMC3476454

[131] Straus SE, Johnson MO, Marquez C, Feldman MD. Characteristics of successful and failed mentoring relationships: a qualitative study across two academic health centers. Acad Med. 2013;88:82–9. doi: 10.1097/ACM.0b013e31827647a0 23165266 PMC3665769

[132] Byars-Winston A, Womack VY, Butz AR, McGee R, Quinn SC, Utzerath E, et al. Pilot study of an intervention to increase cultural awareness in research mentoring: Implications for diversifying the scientific workforce. J Clin Transl Sci. 2018;2:86. doi: 10.1017/cts.2018.25 30338131 PMC6191051

[133] Bond KS, Jorm AF, Kitchener BA, Reavley NJ. Mental health first aid training for Australian medical and nursing students: an evaluation study. BMC Psychology. 2015;3:1–9. doi: 10.1186/s40359-015-0059-2 25914827 PMC4399395

[134] Dutt K. Addressing racism through ownership. Nat Geosci. 2021;14:58–8.

